# I Wouldn’t Search That With My Mobile Phone: Credibility and Trust in OHIRs Among Lower-Income Black Older Adults

**DOI:** 10.1093/geroni/igab046.1960

**Published:** 2021-12-17

**Authors:** Christina Harrington, Amanda Woodward

**Affiliations:** 1 DePaul University, Chicago, Illinois, United States; 2 Michigan State University, East Lansing, Michigan, United States

## Abstract

Online health information resources (OHIRs) such as conversational assistants and smart devices that provide access to consumer health information in the home are promoted as viable options for older adults to independently manage health. However, there is question as to how well these devices are perceived to meet the needs of marginalized populations such as lower-income Black older adults who often experience lower digital literacy or technology proficiency. We examined the experiences of 34 lower-income Black older adults aged 65-83 from Chicago and Detroit with various OHIRs and explored whether conversational resources were perceived to better support health information seeking compared to traditional online web searching. In a three-phase study, participants tracked their experiences with various OHIRs and documented health-related questions in a health diary. Participants were then interviewed about their diaries in focus groups and semi-structured interviews, followed by a technology critique and co-design session to re-envision a more usable and engaging conversational device. We present preliminary results of the themes that emerged from our analysis: cultural variables in health information seeking practices, perceptions of credibility, likelihood of use, and system accessibility. Participants indicated that their trust of different resources depended on the type of information sought, and that conversational assistants would be a useful resource that require less technology proficiency, even among those with lower e-health literacy. Although our findings indicate that familiarity and trust were salient constructs associated with perceptions of OHIRs, these devices may address digital literacy and technology familiarity with certain design considerations.

